# Analogue of Electromagnetically Induced Transparency in an All-Dielectric Double-Layer Metasurface Based on Bound States in the Continuum

**DOI:** 10.3390/nano11092343

**Published:** 2021-09-09

**Authors:** Fengyan He, Jianjun Liu, Guiming Pan, Fangzhou Shu, Xufeng Jing, Zhi Hong

**Affiliations:** Centre for THz Research, China Jiliang University, Hangzhou 310018, China; S1901081112@cjlu.edu.cn (F.H.); jianjun@cjlu.edu.cn (J.L.); gmpan@cjlu.edu.cn (G.P.); fzshu@cjlu.edu.cn (F.S.); jingxufeng@cjlu.edu.cn (X.J.)

**Keywords:** electromagnetically induced transparency, bound states in the continuum, double-layer metasurface

## Abstract

Bound states in the continuum (BICs) have attracted much attention due to their infinite Q factor. However, the realization of the analogue of electromagnetically induced transparency (EIT) by near-field coupling with a dark BIC in metasurfaces remains challenging. Here, we propose and numerically demonstrate the realization of a high-quality factor EIT by the coupling of a bright electric dipole resonance and a dark toroidal dipole BIC in an all-dielectric double-layer metasurface. Thanks to the designed unique one-dimensional (D)–two-dimensional (2D) combination of the double-layer metasurface, the sensitivity of the EIT to the relative displacement between the two layer-structures is greatly reduced. Moreover, several designs for widely tunable EIT are proposed and discussed. We believe the proposed double-layer metasurface opens a new avenue for implementing BIC-based EIT with potential applications in filtering, sensing and other photonic devices.

## 1. Introduction

The analogue of electromagnetically induced transparency (EIT) has attracted much attention since it was realized in metamaterials (MMs) [1,2,3]. The key to realize EIT in MMs is the optical near-field coupling between two resonance modes, which includes the following two ways: bright–dark mode coupling [2,3,4,5,6,7,8] and bright–bright mode coupling [9,10,11,12,13], where the bright mode or dark mode refers to whether a resonance can be directly excited by the incident electromagnetic waves or not. In order to obtain a high-Q EIT, the two resonances need to have a small detuning and large Q contrast [14]. In recent years, the bound state in continuum (BIC) has proved to be an effective method for achieving a high-Q resonance.

The bound state in the continuum lies inside the continuum and coexists with extended waves, but it remains perfectly confined without any radiation [15,16,17,18,19]. In fact, due to the finite extent of structures, material absorption and other external disturbances, ideal (or dark) BICs collapse to a Fano resonance with a finite Q factor, which is called quasi-BIC [20]. At present, a large number of Fano resonances with high Q factors have been obtained through quasi-BIC in the fields of photonic crystals [21,22,23,24], gratings [25,26], waveguides [27] and MMs [28,29]. There are three main types of BIC in MMs: symmetrically protected BICs (S-P BICs) [28,29,30,31,32,33,34], Friedrich–Wintgen BICs (F–W BICs) [35,36,37,38,39] and topologically protected BICs (T-P BICs) [40]. The coupling coefficient could vanish due to the symmetry reason when the spatial symmetry of the mode is incompatible with the symmetry of the of outgoing radiating waves, such a kind of BIC is called S-P BIC [37]. If two resonances pass each other as a function of a continuous parameter, then interference causes an avoided crossing of the resonance positions and at one particular set of the parameters, one resonance has an exactly vanishing width and, hence, becomes an F–W BIC [35].

Recently, a high-Q factor EIT was realized based on an S-P quasi-BIC in a dielectric MM in the case of oblique incidence [41]; however, there are no reports on an ideal BIC-based EIT. Because the commonly used S-P BIC is a longitudinal dipole BIC, for an ideal S-P BIC, it cannot be coupled with a low-Q transverse dipole resonance in the case of normal incidence [41]. On the contrary, the F–W BIC in MMs is usually a transverse dipole BIC [36,37,38], which is easy to be coupled with a low-Q transverse dipole resonance, but it is very sensitive to the structural parameters of MMs. Therefore, it is often difficult to eliminate the detuning between the two coupled F–W BIC and low-Q resonance in single-layer MMs. For double-layer MMs, not only can the two resonances be independently designed, but the near-field coupling of them can also be effectively manipulated by adjusting the relative displacement or distance of the two structures [42,43,44,45], which provides the possibility for the realization of an ideal BIC-based EIT. However, there is no research work reported on this issue yet.

In this paper, we realized and numerically studied an ideal toroidal dipole (TD) BIC-based EIT in the near-infrared range (NIR) in an all-dielectric double-layer metasurface, consisting of a one-dimensional (1D) silicon rod metasurface (RMS) and two-dimensional disk metasurface (DMS). Thanks for the proposed unique 1D–2D double-layer metasurface, a robust high-Q EIT is realized by near-field coupling of a bright electric dipole resonance (ED) of the DMS and a dark TD-BIC of the RMS. The influences of the coupling distance and relative displacement between the double-layer structures on the EIT performance are analyzed. In addition, several methods for achieving a widely tunable EIT are discussed.

## 2. Silicon Rod Metasurface Supporting F–W BIC

**Figure 1:**
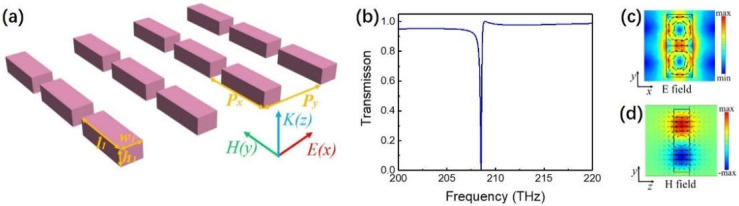
(**a**) A high-Q 2D RMS consisting of a silicon rod array surrounded by air. The length, width and height of the rod are represented by *l*_1_, *w*_1_, and *h*_1_, and the periods of the unit cell in the *x* and *y* directions are *P_x_* = *P_y_* = 900 nm. (**b**) Transmission of 2D RMS when *l*_1_ = 800 nm, *w*_1_ = 335 nm and *h*_1_ = 200 nm. (**c**) Near-field electric field diagram and displacement current (arrow) distribution at the resonance. (**d**) Near-field magnetic field diagram at the resonance.

We began our investigation from a typical 2D RMS supporting a high-Q TD resonance [38,44,46], as shown in Figure 1a. The length, width and height of the rod are represented by *l*_1_, *w*_1_ and *h*_1_, respectively. The periods of the unit cell in the *x* and *y* directions are *P_x_* = *P_y_* = *P* = 900 nm. The permittivity of silicon was set to be 11.9 in simulation. Numerical simulations were carried out by using a commercial finite element frequency domain solver in COMSOL Multiphysics (COMSOL Inc., Stockholm, Sweden). We calculated the transmission of the 2D RMS in the frequency range of 200–220 THz when *l*_1_ = 800 nm, *w*_1_ = 335 nm and *h*_1_ = 200 nm, as shown in Figure 1b. It can be seen from the figure that there was a sharp Fano resonance at 208.5 THz, and its Q value was 1.37 × 10^3^ calculated by the Fano fitting formula [37]. In order to understand the micro properties of this resonance, we used the multipole decomposition calculation in Cartesian coordinates to obtain the contribution of the scattering power of the resonance (not shown in the figure). At the resonance, the TD had the highest scattering power, which was seven times larger than the second largest magnetic quadrupole. The electric and magnetic near-field distributions at the resonance shown in Figure 1c,d also verified the TD resonance: the displacement current shown in Figure 1c formed clockwise and counter clockwise circular loops in the upper and lower parts of the rod, which produced a head-to-tail magnetic moment in the *y–z* plane shown in Figure 1d, indicating that this was a transverse TD along the *x* direction.

**Figure 2:**
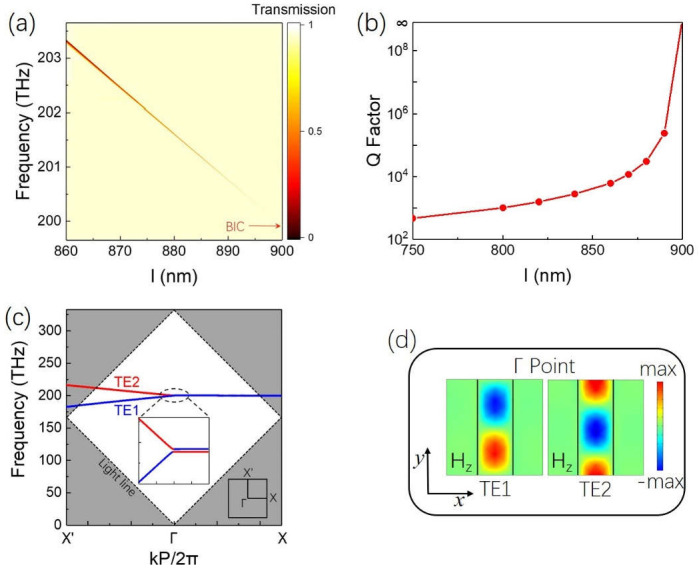
(**a**) Map of transmission spectra for 2D RMS by sweeping length *l*_1_ from 860 to 900 nm, where width *w*_1_ (335 nm) and height *h*_1_ (200 nm) were kept constant. (**b**) Q factor with respect to length *l*_1_. (**c**) Numerically simulated band structure. The bands under consideration were above the light cone. The lower right inset shows the first Brillouin zone; the middle inset is an enlarged view near the Γ point. (**d**) Magnetic field distribution of TE1 and TE2 at Γ point.

The influence of the length *l*_1_ on the TD resonance is shown in Figure 2a. When *l*_1_ increased from 860 nm to 900 nm, the TD resonance red-shifted and gradually narrowed until it disappeared, i.e., the Q factor of the TD resonance shown in Figure 2b rose up quickly and became infinite when *l*_1_ = *P_y_*, which is a typical feature of BIC [19]. Thus, we calculated the dispersion curves of the first Brillouin zone in the ΓX and ΓX’ directions when *l*_1_ = 900 nm. The calculated band structure of the lattice is shown in Figure 2c, where we focused on the two TE bands (TE1 and TE2) above the light cone. The corresponding magnetic field distributions (Hz) are shown in Figure 2d, and the eigenmode corresponding to the TD resonance was TE1. Due to the strong coupling of the TE1 and TE2, the avoiding crossover behavior occurred at the Γ point, resulting in the vanishing of TE1 with an infinite Q factor. Therefore, the F–W BIC condition was satisfied, and the TD-BIC at 200 THz was achieved [35,38]. In fact, when *l*_1_ = *P_y_*, the 2D RMS became 1D BIC-RMS; when *l*_1_ < *P_y_*, the TD-BIC collapsed into a high-Q Fano resonance, i.e., quasi-BIC.

## 3. BIC-Based EIT in 1D–2D Double-Layer Metasurface

### 3.1. Structure Design

**Figure 3:**
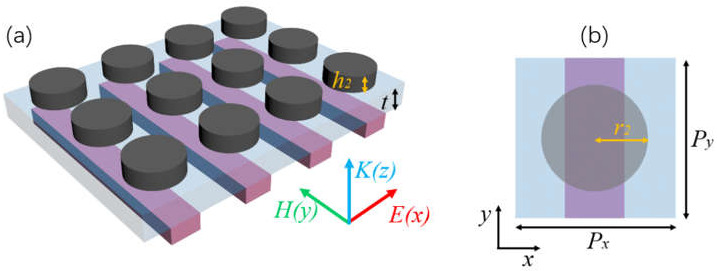
(**a**) Schematic diagram of a double-layer R-DMS. The 2D DMS and 1D RMS were on the top and bottom of a dielectric layer with thickness *t*. (**b**) Top view of the unit cell. Geometric parameters of the disk were *r*_2_ = 328 nm and *h*_2_ = 200 nm.

Since the TD-BIC cannot be directly excited by the normal incident wave in a single-layer RMS, we proposed a double-layer metasurface shown in Figure 3a and demonstrated the realization of EIT by coupling a bright ED mode to the dark TD-BIC. The 2D silicon disk metasurface (DMS) and the 1D BIC-RMS were on the top and bottom of a dielectric layer with thickness t, forming the double-layer metasurface R-DMS. The relative position of the rod and disk in the unit cell is shown in Figure 3b. The disk had radius *r*_2_ = 328 nm and height *h*_2_ = 200 nm; the middle dielectric layer had *ε* = 2.13.

**Figure 4:**
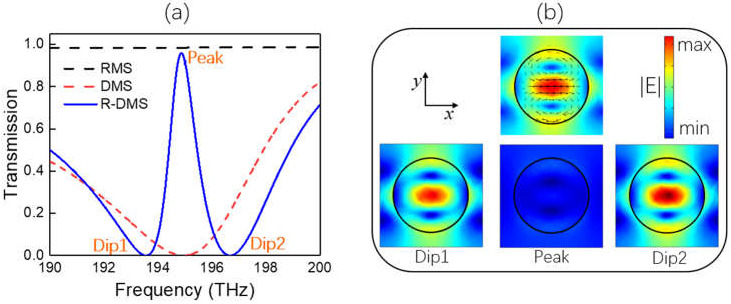
(**a**) Transmission curves for individual 2D DMS (red dashed line), individual 1D RMS (black dashed line), and double-layer R-DMS (blue solid line) when *t* = 600 nm. Dip1, Dip2 and Peak refer to frequencies of the two dips and peak in the EIT transparency window, respectively. (**b**) The first row displays the electric near-field distribution and displacement current at the resonance of 195 THz for the individual DMS, the second row is the electric near-field distributions in the disk at Dip1, Peak and Dip2 after being coupled.

The calculated transmission of the individual DMS with a dielectric layer is shown in a red dashed line in Figure 4a. There was a wideband ED resonance centered at 195 THz with a Q factor of 23. The first row in Figure 4b displays the electric near-field distribution and displacement current in the disk at the resonance, which shows an obvious ED in the *x*-direction in the center of the disk. In addition, two circular displacement current loops with opposite directions were formed in the upper and lower parts of the disk, which was similar to that in the rod of RMS in Section 2, i.e., TD moment along the *x*-direction also made a significant contribution to the resonance. The multipole decomposition result (not shown here) shows that the scattering power of the ED at the resonance was the highest and dominant, which was four times larger than that of the TD moment.

When the 2D DMS and 1D RMS formed a double-layer R-DMS, we found that the bright ED resonance of the DMS could be easily and strongly coupled to the dark TD-BIC of the RMS, leading to an EIT transparency window at 194.9 THz with a bandwidth of 1.1 THz, as shown in a blue solid line in Figure 4a. The black dashed line in the figure represents the transmission of the individual 1D RMS with the dielectric layer, the TD-BIC red-shifted to 194.9THz compared to that of 200 THz in Figure 1 due to the influence of the dielectric layer; thus, the detuning between the ED and TD-BIC was small. As a result, the EIT transparency window had a good symmetry. The near-field coupling between the two modes could also be verified from the electric near-field distributions in the disk at Dip1, Peak and Dip2 shown in Figure 4b: the ED in the disk at Peak became very weak due to destructive interference, and it is much weaker than that at Dip1 and Dip2, while the electric and magnetic near-field distributions in the rod (not shown in the Figure) were quite similar to those in Figure 1 because of the excitation of the TD-BIC.

### 3.2. Structural Parameter Analysis

**Figure 5:**
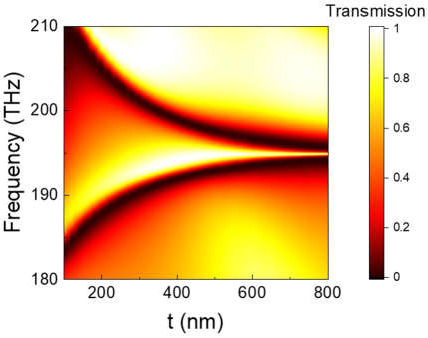
Map of transmission spectra for R-DMS by sweeping coupling distance *t* from 100 to 800 nm, when *l*_1_ = 900 nm, *w*_1_ = 335 nm.

The near-field coupling of the two resonances would largely determine the EIT performance, which could be manipulated by the coupling distance (thickness *t*) or the relative position between the two structures. Figure 5 shows the EIT transmission spectra when the coupling distance changed from 100 to 800 nm. When *t* was small (100–400 nm), the near-field coupling of the ED and TD-BIC was very strong, resulting in a wideband transparency window. Because the ED resonance had a certain degree of asymmetry, the EIT transparency window was asymmetric as well. As *t* increased from 400 nm to 800 nm, the near-field coupling between the two resonances became weaker and weaker. Therefore, the EIT transparency window became narrower and more symmetrical; its Q value rapidly increased from 20 to 816, and, meanwhile, the peak of the EIT maintained a large value over 0.93.

**Figure 6:**
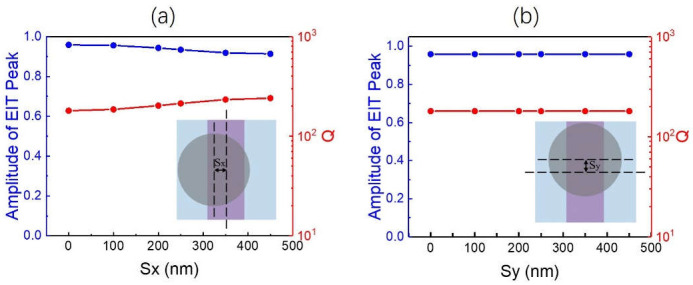
Influence of the relative displacement between the disks and rods in the x-direction *S_x_* (**a**) and *y*-direction *S_y_* (**b**) on the EIT performance when *P_x_* = *P_y_* = 900 nm, *w*_1_ =335 nm, *r*_2_ = 328 nm and *t* = 600 nm.

We also investigated the dependence of the EIT performance on the relative displacement between the RMS and DMS, as shown in Figure 6. The relative displacement between the disks and rods in the *x*, *y* direction is represented by *S_x_*, and *S_y_*, respectively. When *S_x_* changed from 0 nm to the full range of 450 nm, a robust EIT was achieved due to a good coupling ability between the ED and TD-BIC; the peak transmittance of the EIT only decreased from 0.96 to 0.91, and the Q factor increased from 180 to 241, as shown in Figure 6a. Moreover, owing to the special 1D–2D combination of the double-layer R-DMS, *S_y_* had no impacts on the EIT as shown in Figure 6b. Considering the EIT conventionally realized by the coupling of the two resonances in a 2D–2D double-layer metasurface (including 2D–2D R-DMS here) [42,44], not only the length of the rod, but also the relative displacement *S_y_* would have a great influence on the EIT performance. The greatly reduced sensitivity of the proposed TD-BIC-based EIT to the relative displacement would ease the fabrication of the double-layer metasurface.

### 3.3. Widely Tunable EIT Based on TD-BIC

**Figure 7:**
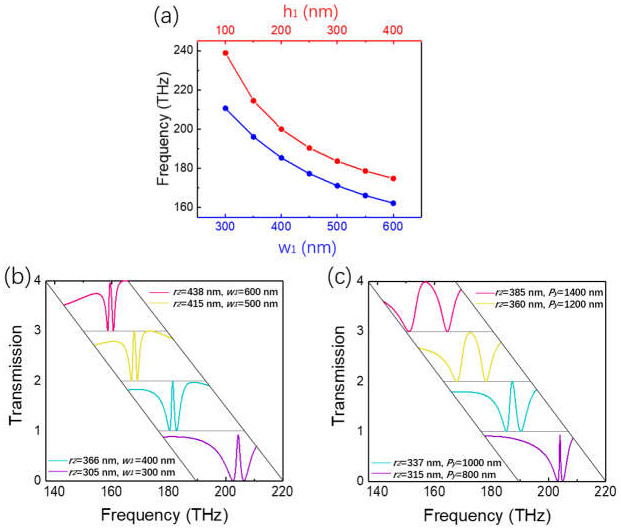
(**a**) Resonance frequencies of the TD-BIC with respect to rod width *w*_1_ when *h*_1_ = 200 nm (blue), and with respect to height *h*_1_ when *w*_1_ = 335 nm (red). (**b**) EIT tuning characteristics obtained by changing disk radius *r*_2_ and rod width *w*_1_. (**c**) EIT tuning characteristics obtained by changing disk radius *r*_2_, lattice constant and let *P_x_* = *P_y_* for simplicity. The thickness *t* was fixed of 600 nm and *h*_1_ = *h*_2_ = 200 nm.

The EIT working in a large frequency range is important for real applications. In order to achieve a widely tunable TD-BIC-based EIT, it is necessary to change the size of the rod so that the TD-BIC can be tuned in a wide frequency range; at the same time, the frequency of the ED resonance needs to be adjusted to roughly match the TD-BIC as well. We used the same eigenmode analysis as in Section 2 to study the dependence of the TD-BIC on the rod’s geometric parameters. The results showed that for the fixed rod length *l*_1_ = *P_y_* = 900 nm, when *w*_1_ varied in the range of 300–600 nm or *h*_1_ in the range of 100–400 nm, the conditions of F–W BIC for the TD resonance were all satisfied; the frequency of the TD-BIC with respect to *w*_1_ and *h*_1_ are displayed in Figure 7a. As *w*_1_ increased from 300 nm to 600 nm, the frequency of the TD-BIC decreased from 210.7 THz to 162.2 THz (from 204.4 THz to 159.5 THz when rod array with dielectric layer). Similarly, as *h*_1_ increased from 100 nm to 400 nm, the TD-BIC resonance also showed a downward trend, from 238.9 THz to 174.9 THz. Obviously, the TD-BIC was very sensitive to the rod’s width and height. Meanwhile, the ED was very sensitive to the disk radius *r*_2_; when *r*_2_ increased from 305 nm to 438 nm, the resonance frequency of the ED decreased from 205.7 THz to 162 THz. Therefore, a widely tunable EIT can be achieved via near-field coupling of the two small, detuned resonances by changing the disk radius and rod width, as shown in Figure 7b. Four EIT transparency windows were obtained at different rod widths of 600 nm, 500 nm, 400 nm and 300 nm; the corresponding disk radii were 438 nm, 415 nm, 366 nm and 305 nm, respectively. The TD-BIC-based EIT tuned from 159.5 THz to 204.4 THz, with a large peak value over 0.9 and a small variation bandwidth (1.17 ± 0.18 THz).

Another interesting way to achieve a widely tunable EIT is by changing the disk radius *r*_2_ and lattice constant *P_y_*. Actually, for the 1D RMS, the variation of *P_y_* did not change the RMS at all. However, it was meaningful for the tuning of the dark TD-BIC. In this way, a widely tunable EIT was achieved as shown in Figure 7c. When *P_y_* increased from 800 nm to 1400 nm, and the disk radius *r*_2_ varied from 315 nm to 385 nm to make the detuning of the two resonances small, as a result, the EIT with high peak transmittance tuned from 203.9 THz to 156.8 THz; nevertheless, the bandwidth of the EIT increased largely from 0.47 THz to 8.46 THz. This was because as the radius of the disk increased, the near-field coupling between the ED and TD-BIC became stronger and stronger, resulting in a substantial increase in the bandwidth of the EIT.

Considering the fabrication of the device we designed, in the existing technology, for the double-layer all-dielectric metasurface of the optical waveband, silicon can be deposited by using plasma-enhanced chemical vapor deposition on both sides of the substrate, and then the nano pattern can be manufactured by electron-beam lithography and dry etching [47,48]. Due to the very thin dielectric layer of the structure, an additional layer of substrate is required for a real device, which may have a certain impact on the simulation results. When the proposed structure was extended to the terahertz band, the coupling distance could be larger than 500 μm [49], no additional substrate was needed and the device could be easily fabricated by photolithography and deep etching [38].

## 4. Conclusions

In summary, we proposed and numerically demonstrated the realization of high-Q an EIT in NIR by coupling the bright ED resonance to the dark TD-BIC in an all-dielectric double-layer metasurface. Owing to the unique 1D–2D combination of the double-layer metasurface, the sensitivity of the EIT to the relative displacement between the double-layer structures was greatly reduced. When the relative displacement changed in its full range, the peak value of the robust EIT remained above 0.90. As the coupling distance increased, the Q factor of the EIT reached 816. Additionally, a much higher Q value of the EIT could be expected by weakening the near-field coupling of the two resonances in all-dielectric or plasmonic-dielectric hybrid double-layer metasurfaces. In addition, several methods for widely tunable EIT were proposed and discussed, and the EIT with a tunable range of ~45 THz and a small variation bandwidth (1.17 ± 0.18 THz) was demonstrated by changing the disk radius and rod width. We believe the proposed double-layer metasurface provides a new platform for implementing BIC-based EIT and can be extended to other electromagnetic waves such as microwaves and the terahertz band.

## Data Availability

The data are available on reasonable request from the corresponding author.
